# The South African Rugby Injury and Illness Surveillance and Prevention Project (SARIISPP): SARU Girls’ Youth Week Injury Surveillance Report 2023

**DOI:** 10.17159/2078-516X/2025/v37i1a23951

**Published:** 2025-12-15

## Executive Summary

As part of the South African Rugby (SARU) Injury and Illness Surveillance and Prevention Project (SARIISPP), SARU records and investigates injury data from their annual SARU Youth Week tournaments. The BokSmart National Rugby Safety Programme has gathered and analysed these data yearly since 2015 for the SARU Girls’ Youth Weeks.

No Gu16W and Gu18W tournaments were held in 2020 and 2021 due to the COVID-19 restrictions. In 2022, the tournament format was amended to allow each age group to play only twice during the week. This was done to allow for a rest day to be inserted between each allocated playing day. The same format was applied in 2023.

This report focuses on the 2023 Girls Rugby tournaments: Girls Under 16 Week (Gu16W) and Girls Under 18 Week (Gu18W). Each tournament consisted of 16 teams and a total of 24 matches were played in 2023. Due to snow, the first day’s matches were cancelled for the Gu16W. Therefore, they only played 8 matches for this tournament. Comparisons between the two SARU Girls’ Youth Week tournaments (Gu16W vs. Gu18W) are made in 2023 and over time between 2015 and 2023. It is worth noting that no Gu16W tournament was held in 2017.

Each medical facility at the SARU Youth Week tournaments has a designated researcher(s) on site, who, together with the tournament medical doctors, records the tournament injury data daily. Over time, this analysis investigates injury patterns in the SARU Girls’ Youth Weeks (Gu16W and Gu18W). Furthermore, the analysis compares the profiles of injured players at each tournament. Throughout the analysis and investigation of these patterns, any areas of concern that potentially require changes to the game, tournament structure, or medical support services are identified. Consequently, injury-specific interventions can be developed and implemented where the evidence supports such a need.

In 2023, there were 56 time-loss injuries for both tournaments (Gu16W and Gu18W) combined, at an average of 70 (52 – 88) injuries per 1000 player hours; data are expressed as mean (95% confidence intervals) injuries per 1000 player hours. The Time-Loss injury incidence for the Gu16W and Gu18W tournaments was 88 (50 – 125) injuries per 1000 player hours and 63 (42 – 83) injuries per 1000 player hours, respectively. Combining the injury incidence data collected over the seven years, there was no significant difference between the two age groups. However, Gu18W had a lower injury incidence compared to Gu16W.

In 2023, *Tackling* (tackler) was the most frequent injury-causing event, followed by *Being Tackled* (ball carrier), and then *Ruck*. *Tackling front-on (regulation)* and *Tackling behind (regulation)* were the most frequent injury-causing mechanisms involved in the Tackler injuries. *Being Tackled front-on (regulation)*, *Being Tackled from behind (regulation)*, and *Being Tackled side-on (regulation)* were equally the most frequent injury-causing mechanisms involved in Ball Carrier injuries. Lastly, *Rucked* was the most frequent injury-causing mechanism in *Ruck* injuries.

The most common injury type for the combined tournaments in 2023 was *Central Nervous System* injuries. No differences were found between tournaments. *Lower Body* was the most common injury location in 2023, accounting for 39% of the injuries, followed by *Head and Neck* injuries. Players who started the match sustained more injuries than those who joined the match as substitutes. Scrumhalves were the player position with the highest normalised injury incidence per player per position across both tournaments. As expected, the incidence of *‘New’* injuries was higher than subsequent ‘*Recurrent’* injuries. Most muscle and joint/ligament injuries were ‘*New’ injuries*, albeit that joint/ligament injuries had proportionately more ‘*Recurrent’* injuries than muscle injuries.

In 2023, 13 concussions were recorded for both tournaments with an incidence rate of 16 (7 – 25) concussions per 1000 player hours. This figure marks a decrease from the record-high rate of concussions recorded in 2022. Furthermore, the act of *Tackling* and *Being Tackled* each contributed to 39% of the events causing concussions.

This report recommends maintaining the current competition format until the Girls’ Youth Game at schools and youth clubs becomes more established, even though there was a decrease in injury and concussion rates in the Gu18W from 2022.

Furthermore, there must be an ongoing emphasis on building confidence and skill in contact among young female rugby players. Progressive and tailored training in tackling and ball-carrying techniques for junior South African female rugby players must continue to be prioritised to develop the necessary contact safety skills, techniques, and required proficiency levels, thereby minimising concussions and general rugby injuries. Introducing the “Preparation for Contact” and “Contact Confident” programmes, freely accessible on the World Rugby Passport education platform and via MyBokSmart on the BokSmart 8 Rugby Safety Course, may assist with the training and contact preparation of junior South African female rugby players. This will over time prove beneficial for reducing the risk of concussions and general musculoskeletal injuries.

**Figure:**
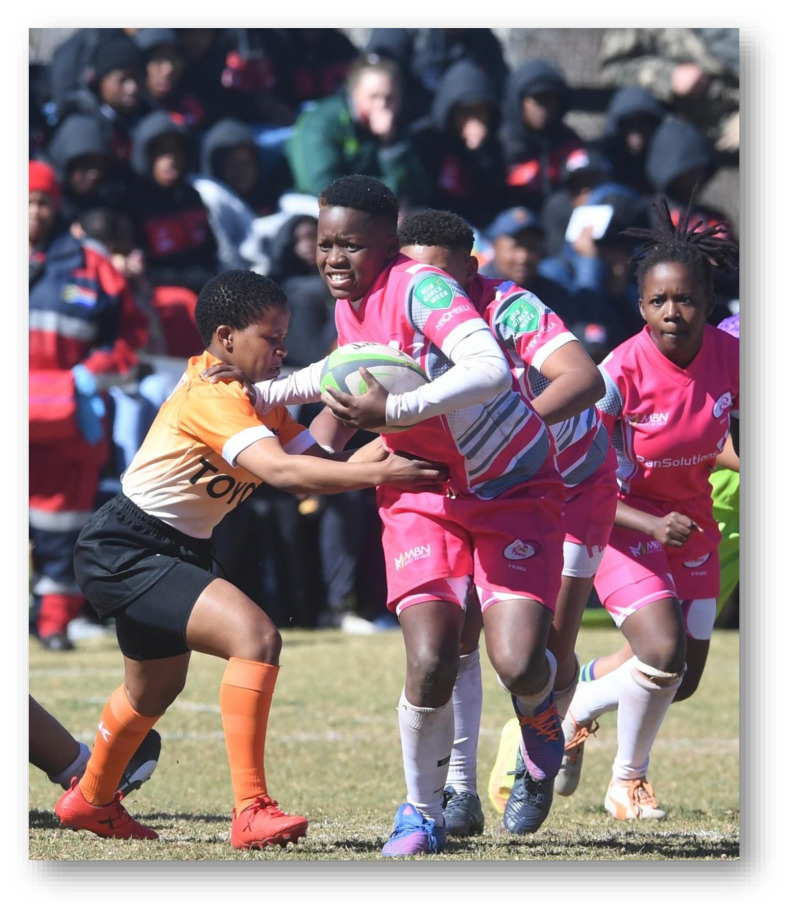

## Definitions

All definitions are originally based on the 2007 consensus statement for injury reporting in rugby union (1) and have since been realigned with the latest International Olympic Committee (IOC) consensus statement for methods of recording and reporting epidemiological data on injury and illness in sport (2).

### MEDICAL ATTENTION INJURY

All injuries seen by the tournament medical doctor or medical support staff were classified as Medical Attention injuries. These injuries are defined by the 2007 statement as an “*injury that results in a player receiving medical attention”* (1) and by the more recent IOC statement as *“a health problem that results in an athlete receiving medical attention”* (2).

### TIME-LOSS INJURY

Medical Attention injuries were further categorised as Time-Loss injuries, where appropriate, and defined by the 2007 statement as “*an injury that results in a player being unable to take a full part in future rugby training or match play*” (1). The IOC definition is *“a health problem that results in a player being unable to complete the current or future training session or competition”* (2). For clarity, this means an injury sustained by a rugby union player during a match or training session that prevented or would have prevented the player from taking full part in all rugby training activities and/or match play for more than 1 day following the day of injury, irrespective of whether match or training sessions were scheduled (3).

### INJURY RATE

This report defines an injury rate as the number of injuries per 1000 player exposure hours. This method of expressing injury rate has been used in previous Youth Week reports and other international literature, making comparisons easy. Moreover, the injury rate is expressed as a mean with 95% confidence intervals. A 95% confidence interval around a mean value indicates a 95% chance (i.e., very high chance) that the true value falls within this range. In this report, we present the 95% confidence intervals assuming a normal distribution of the data and use the approach of examining the overlap of the confidence intervals to determine whether the injury incidences are significantly different; if the range of confidence interval values of two comparisons does not overlap, there is a strong chance (95%) that their injury rates are different from each other. We have opted for this method because it is easy to use, conservative and less likely to produce false positive results (4).

### NEW, SUBSEQUENT AND RECURRENT INJURIES

In the 2023 Girls’ Youth Week, a ‘*New Injury’* was defined as when a player sustained her first injury. Any injury the *same* player sustained after this initial injury was defined as a *‘Subsequent Injury’.*

According to the IOC statement, any subsequent injury to the same site and of the same type is referred to as a ‘*Recurrence’* if the index injury was fully recovered before reinjury, and as an *‘Exacerbation’* if the index injury was not yet fully recovered (2).

To provide more detail on subsequent injuries for practitioners, one can further categorise the subsequent injuries into one of four groups:

- Different site - Different type
- Different site - Same type
- Same site - Different type
- Same site - Same type

According to the 2007 Consensus Statement for rugby, any subsequent injury classified as ‘Same site - Same type’ was a *‘Recurrent injury’* (1).

**Figure:**
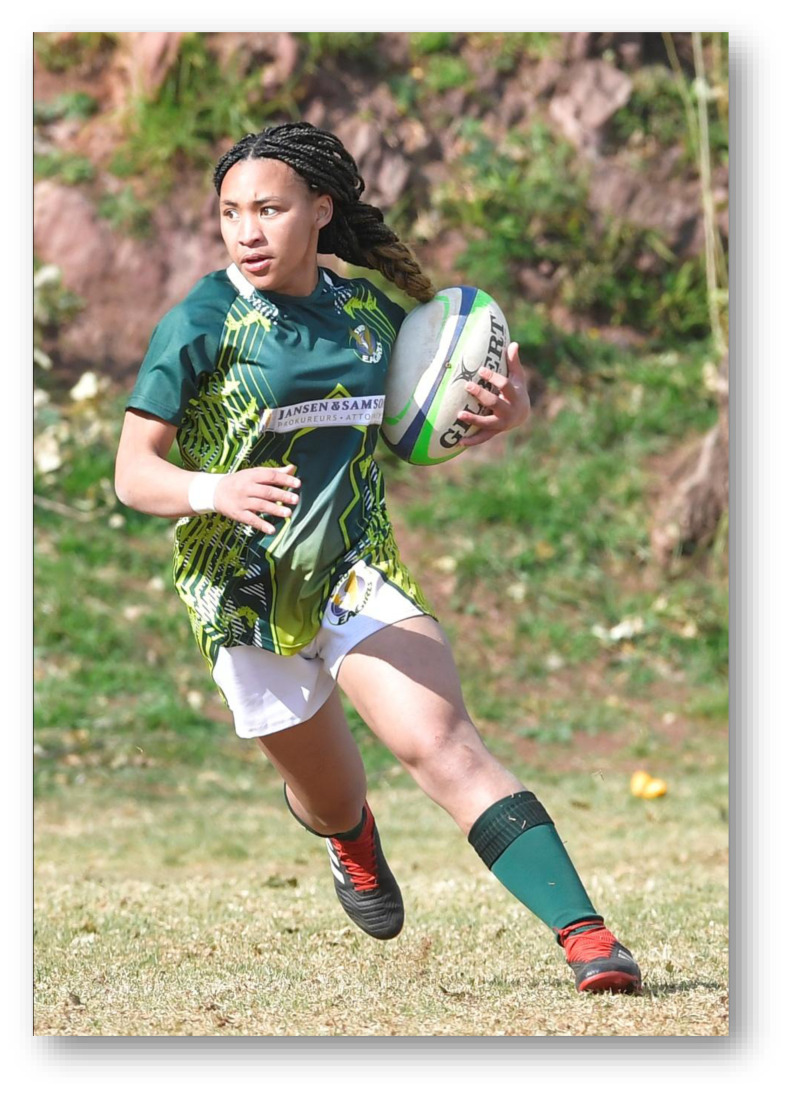

## Key Findings

### Injury Incidence

**Figure 1:**
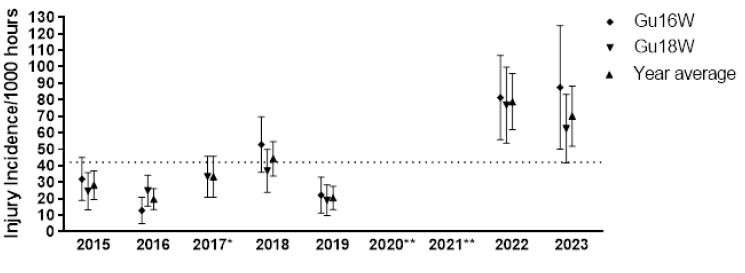
Incidence/1000 player hours and 95% confidence intervals of injuries for the SARU Girls’ Youth Week tournaments from 2015 to 2023. The dotted line reflects the average incidence for all tournaments over all the included years. *No Gu16W tournament was held in 2017. **No Gu16W and Gu18W tournaments were held in 2020 and 2021 due to COVID-19 restrictions.

In 2023, thirty-two teams participated in the SARU Girls’ Youth Week tournaments (Gu16W = 16 teams, Gu18W = 16 teams). A total of 77 Medical Attention injuries were recorded during the tournaments. Fifty-six (56) of these were Time-Loss injuries (73%). The combined tournaments’ injury incidence and 95% confidence intervals for all Medical Attention injuries were 96 (75 – 118) injuries/1000 player hours, and for Time-Loss injuries were 70 (52 – 88) injuries/1000 player hours. No significant difference in injury incidence was found between the two SARU Girls’ Youth Week tournaments in 2023 (Table 1). Table 2 represents the Medical Attention and Time-Loss injuries per match and per hour of match play across both tournaments. On average, there were approximately 2 time-loss injuries for every match played (or 7 injuries for every 3 matches played). Figure 1 shows the pattern of incidence/1000 player hours and 95% confidence intervals of Time-Loss injuries for each tournament (2015 to 2023). Time-Loss incidence for the Gu16W and Gu18W tournaments and the combined incidence of both tournaments is significantly greater in 2022 and 2023 compared to the tournaments’ historical average. It must be noted that the Gu16W tournament in 2017 did not take place, and neither did Gu16W and Gu18W in 2020 and 2021 due to COVID-19 restrictions (Figure 1).

**Figure 2:**
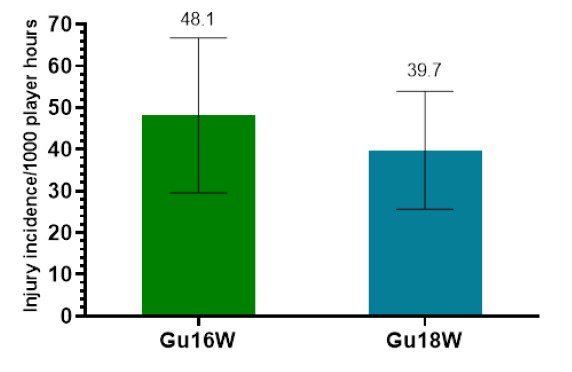
Injury incidence/1000 player hours and 95% confidence intervals for the SARU Girls’ U16 (Gu16W) and U18 (Gu18W) Youth Week tournaments from 2015 to 2023. The number above each bar represents the average injury incidence/1000 player hours over the time-period of data collection.

Only Time-Loss injuries were analysed further in this report (n = 56). Figure 2 demonstrates a slightly lower combined injury incidence in Gu18W (2015 to 2023) compared to Gu16W. These two tournaments showed no significant differences in their injury incidence rates.

**Figure:**
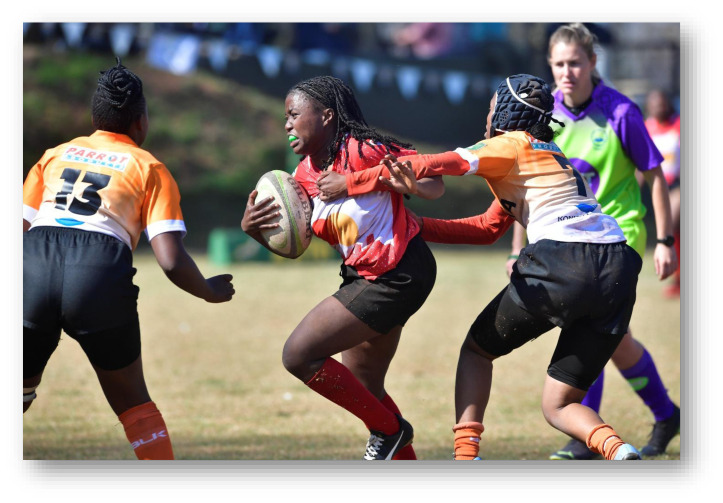

#### Injury Incidence Trends

##### Girls U16 Week (Gu16W)

**Figure 3:**
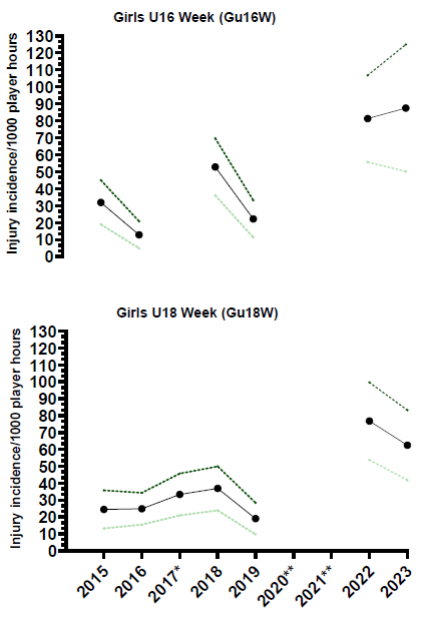
Time-Loss injury incidence for each SARU Girls’ Youth Week tournament per year from 2015 to 2023, including the upper and lower 95% Confidence Intervals (95% CI). *No Gu16W tournament was held in 2017. **No Gu16W and Gu18W tournaments were held in 2020 and 2021 due to COVID-19 restrictions.

The Gu16W tournament was not held in 2017, 2020 and 2021; therefore, the trendline could not be calculated accurately and has been excluded. The highest injury incidence was recorded in 2023, with a large upward spike in injuries first demonstrated between 2019 and 2022. There was only a minor, non-significant increase from 2022 to 2023 (Figure 3).

##### Girls U18 Week (Gu18W)

The Gu18W tournament was not held in 2020 and 2021; therefore, the trendline could not be accurately calculated and has been excluded. After a slight decrease in 2019, as with the Gu16W, there was a significant increase in injury incidence in 2022 at the Gu18W (Figure 3). The injury incidence in 2019 was the lowest in the initial 5 years of the study, and the increase in 2022 was the highest. In 2023, the injury incidence decreased again, although it was not significantly different from that of 2022 (Figure 3).

### Injury Event

In 2023, the *Tackler* role was the event associated with the most injuries throughout the tournaments (41%, n = 23), followed by the *Ball Carrier* role (34%, n = 19) and then the *Ruck* (18%, n = 10). *Tacklers* recorded 29 (17 – 41) injuries/1000 player hours, while *Ball Carriers* had an incidence of 24 (13 – 34) injuries/1000 player hours, with the *Ruck* at 13 (5 – 20) injuries/1000 player hours. In the Gu18W tournament, *Tackler* injuries dominated, while in the Gu16W, *Being Tackled (Ball Carriers)* were the most common injury-causing events (Table 3).

**Figure 4:**
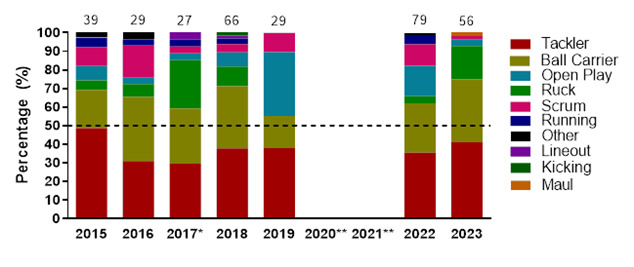
Most common injury-causing events in the SARU Girls’ Youth Week tournaments from 2015 to 2023. (The number above each bar represents the total number of injuries for that year). Missing 2023 data = 0 cases. *No Gu16W tournament was held in 2017. **No Gu16W and Gu18W tournaments were held in 2020 and 2021 due to COVID-19 restrictions.

Figure 4 displays the grouped proportional breakdown of injuries between 2015 and 2023, resulting from the different injury-causing events. The proportions of *Tackler* and *Ball Carrier* injuries fluctuated throughout the years but have remained the most prominent injury-causing events. The Tackle event (both *Tackler* and *Ball carrier* combined) ranged between 55% – 75% (averaging 65%) of all injuries per year in the Girls’ Weeks (both Gu16W and Gu18W) during this period. There was an increase in injuries from the *Ruck* and to the *Ball Carrier* in 2023. While injuries occurring during *Open Play*, decreased between 2022 and 2023 (no tournaments were played in 2020 and 2021).

**Figure 5:**
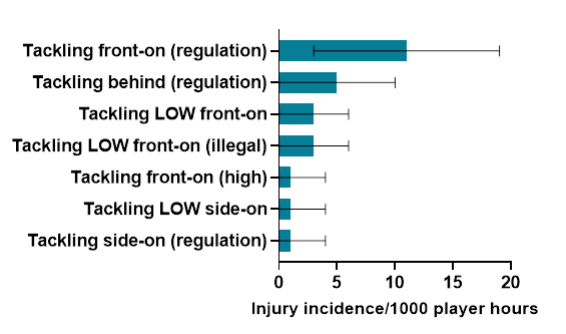
Injury incidence and 95% confidence intervals/1000 player hours of Tackler-related injury mechanisms at the 2023 SARU Girls’ Youth Week Tournaments. Missing 2023 data = 3 cases.

Ranked from highest to lowest, in 2023, *Tackling front-on (regulation)* and *Tackling from behind (regulation)* were the mechanisms that accounted for the most injuries to *Tacklers*. *Tackling front-on (regulation)* accounted for 39% of *Tackler* injuries, with 11.3 (3.9 – 18.6) injuries/1000 player hours (Figure 5).

**Figure 6:**
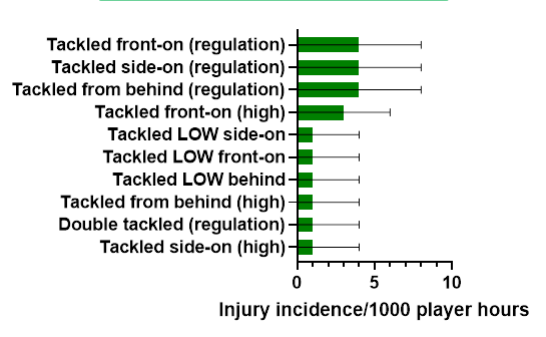
Injury incidence and 95% confidence intervals/1000 player hours of Ball Carrier-related injury mechanisms at the 2023 SARU Girls’ Youth Week Tournaments. Missing 2023 data = 2 cases.

Ranking the mechanisms of injury to *Ball Carrier*s from highest to lowest contributors, in 2023, *Tackled front-on (regulation), Tackled from behind (regulation)* and *Tackled side-on (regulation)* were shared highest, followed by *Tackled front-on (high). Tackled front-on (regulation), Tackled from behind (regulation)* and *Tackled side-on (regulation)* each accounted for 16% of these injuries, with an incidence of 3.8 (0.3 – 8.0) injuries/1000 player hours (Figure 6).

**Figure 7:**
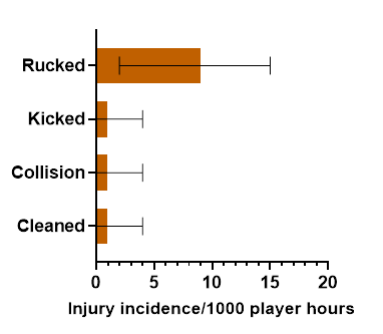
Injury incidence and 95% confidence intervals/1000 player hours for Ruck-related injury mechanisms at the 2023 SARU Girls’ Youth Week Tournaments. Missing 2023 data = 0 cases.

*Rucked* accounted for the highest proportion of *Ruck*-related injuries (70%), with 8.8 (2.3 – 15.2) injuries/1000 player hours (Figure 7).

### Injury Type

In 2023, CNS (Central Nervous System) injuries were the most prevalent type of injury (Table 4). There were no significant differences between the various types of injuries and age groups, nor were there any discrepancies in the combined data across both tournaments.

**Figure 8:**
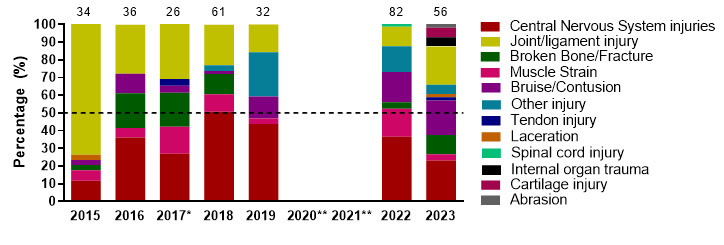
Most common injury types in the SARU Girls’ Youth Week tournaments from 2015 to 2023. (The number above each bar represents the total number of injuries for that year). *No Gu16W tournament was held in 2017. **No Gu16W and Gu18W tournaments were held in 2020 and 2021 due to COVID-19 restrictions. Missing 2023 data = 0 cases.

Figure 8 displays the most common injury types in proportionate distributions per year from 2015 to 2023. The proportion of CNS and muscle injuries decreased from 2022 to 2023, but CNS injuries remain the most common injury type in the SARU Girls’ tournaments over the years. It is worth noting that since the first tournament data was collected in 2015, there has been a slow but consistent reduction in the proportionate distribution of Joint/Ligament injuries until 2022. In 2023, Joint/Ligament injuries increased from 2022, but remained lower than the pre-2018 era. Broken Bone/Fracture injuries also increased from 2022 to 2023.

### Body Location

Injuries were grouped according to the four main body location groups (*Head and Neck; Trunk; Upper Body; Lower Body*) across both tournaments. In 2023, the most common injured body location was *Lower Body* injuries (39%), with 73% of these injuries occurring at the Gu18W tournament. *Lower Body* injuries accounted for an injury incidence of 28 (16 – 39) injuries/1000 player hours (Table 5). The Gu18W had the highest *Lower Body* incidence recorded at 29 (15 – 43) injuries/1000 player hours, but this was not significantly different from the Gu16W tournament. Following *Lower Body* injuries, *Head and Neck* injuries were the second most common. *Head and Neck* accounted for an injury incidence of 21 (11 – 31) injuries/1000 player hours. Gu16W had the highest *Head and Neck* incidence of 29 (8 – 51) injuries/1000 player hours, which was not significantly different to the Gu18W tournament.

**Figure:**
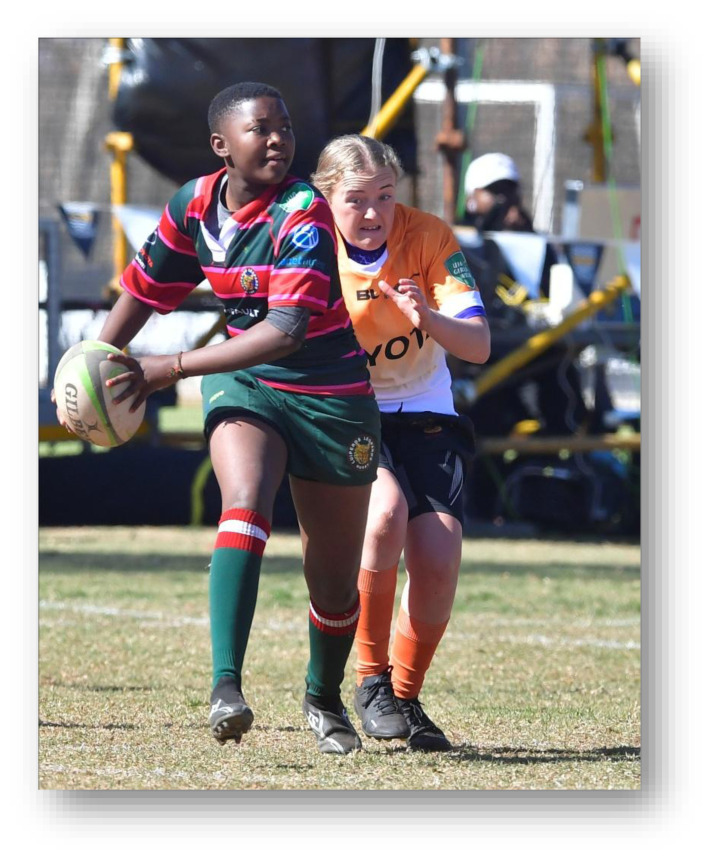

The IOC Consensus statement’s recommended categories of Tissue and Pathology injury data are presented in Table 6 for the 2023 SARU Girls’ Youth Week tournaments (2).

**Figure:**
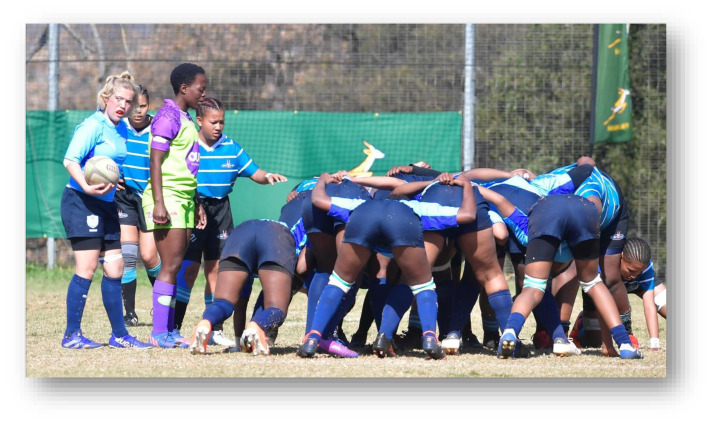

### Match Status

**Figure 9:**
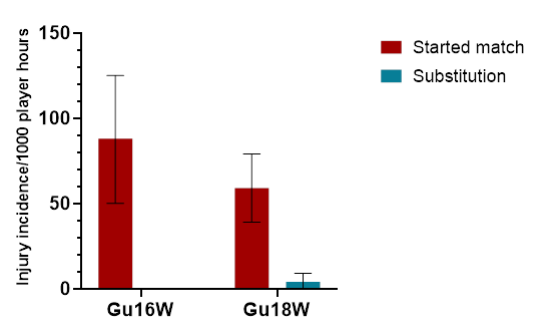
Injury incidence and 95% confidence intervals/1000 exposure hours of players who started the match and those who came on as substitutes in the 2023 SARU Girls’ Youth Week tournaments. Missing 2023 data = 0 cases.

In 2023, there were significantly more injuries to players who started the match (96%) compared to players who joined the match as substitutes (4%) (Figure 9). Injury rates for players who started the match were significantly higher than for players who came on as substitutes in Gu16W and Gu18W. No significant differences were found between tournaments (Table 7).

### New vs. Recurrent

The incidence of *‘New’* injuries in 2023 was 60 (41 – 74) injuries/1000 player hours, significantly higher than ‘*Recurrent’* injuries, which had an incidence of 13 (5 – 20) injuries/1000 player hours.

**Figure 10:**
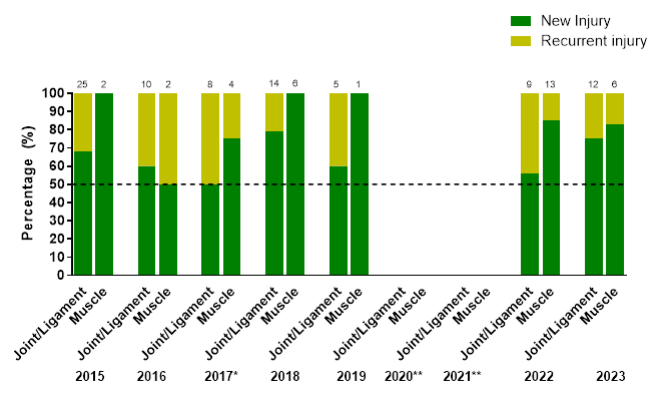
Proportion of New and Recurrent joint/ligament and muscle injuries in the SARU Girls’ Youth Week tournaments from 2015 to 2023. (The number above each bar represents the total number of injuries for that year). *No Gu16W tournament was held in 2017. **No Gu16W and Gu18W tournaments were held in 2020 and 2021 due to COVID-19 restrictions.

Figure 10 illustrates the proportion of *‘New’* and *‘Recurrent’* joint/ligament and muscle injuries across the years (2015–2023). Most muscle and joint/ligament injuries were ‘*New’*, albeit that joint/ligament injuries had proportionately more ‘*Recurrent’* injuries than muscle injuries.

The proportion of *‘New’* joint/ligament injuries increased from 2022 (56%) to 2023 (75%). *‘New’* muscle injuries are similar between 2022 (85%) and 2023 (83%).

The reciprocal percentage ‘*Recurrent’* joint/ligament injuries decreased from 2022 (44%) to 2023 (25%), and ‘*Recurrent’* muscle injuries were similar in 2022 (15%) and 2023 (17%).

### Game Quarter

**Figure 11:**
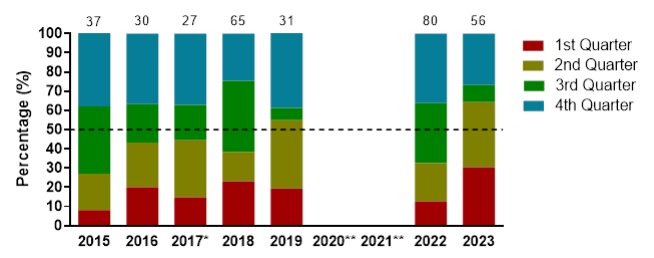
Proportion of injuries occurring in each game quarter in the SARU Girls’ Youth Week tournaments from 2015 to 2023. (The number above each bar represents the total number of injuries for that year). Missing 2023 data = 0 cases. *No Gu16W tournament was held in 2017. **No Gu16W and Gu18W tournaments were held in 2020 and 2021 due to COVID-19 restrictions.

In 2023, most injuries occurred in the 2^nd^ quarter (34%) followed by the 1^st^ quarter (30%) with an incidence of 24 (13 – 34) injuries/1000 player hours and 21 (11 – 31) injuries/1000 player hours, respectively. Injuries in the 3^rd^ match quarter decreased proportionately from 2022 to 2023, although this was not significantly different (Figure 11).

**Figure:**
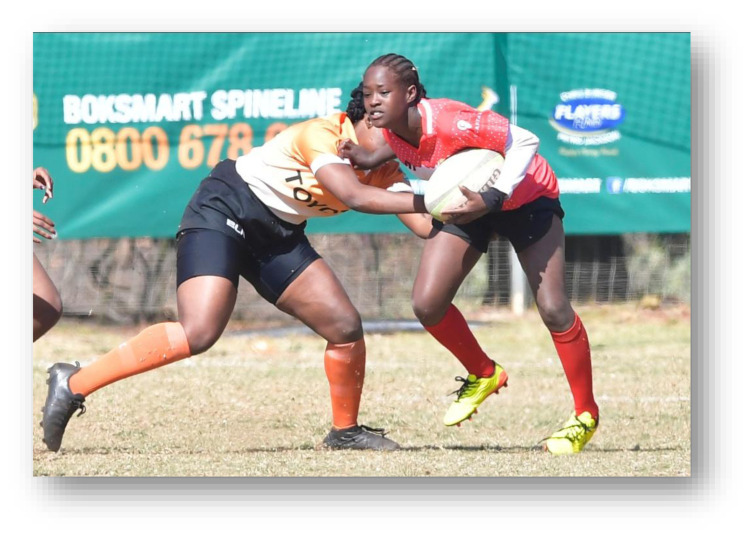

### Player positions

**Figure 12:**
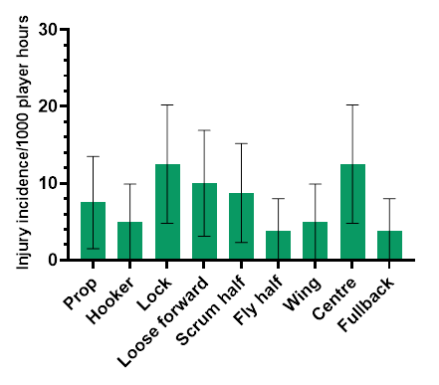
Absolute injury incidence and 95% confidence intervals/1000 player hours per positional grouping in the combined 2023 SARU Girls’ Youth Week Tournaments. Missing 2023 data = 1 case.

Centres and locks had an *absolute* injury incidence of 12.5 (4.8 – 20.2) injuries/1000 player hours (Figure 12). *Absolute* incidence refers to the incidence or rate of injury in a player’s positional grouping, e.g., wings, without normalising for the number of players on the field playing in that positional grouping per team, e.g., there are two wings per team on the field. In 2023, the centre and lock positions had the highest *absolute* injury incidence rates across the SARU Girls’ Youth Week tournaments.

The number of injuries was also *normalised* to the number of players per team on the field in a positional grouping. For example: Props = total number of injuries divided by 2, Locks = total number of injuries divided by 2, Loose forwards = total number of injuries divided by 3.

**Figure 13:**
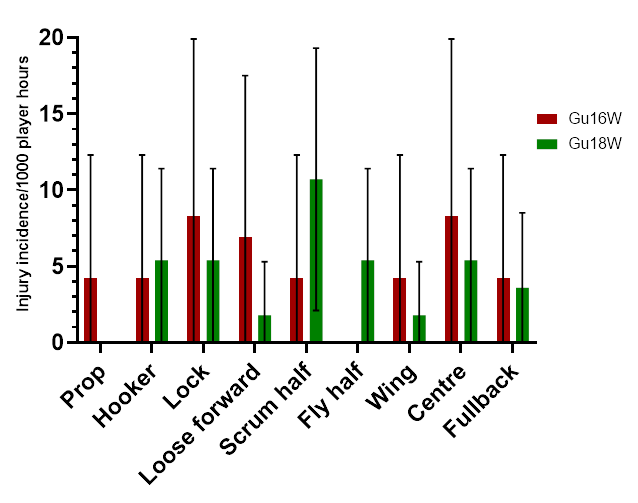
Normalised injury incidence and 95% confidence intervals/1000 player hours per player per position in the two 2023 SARU Girls’ Youth Week Tournaments. Missing 2023 data = 1 case.

**Figure 14:**
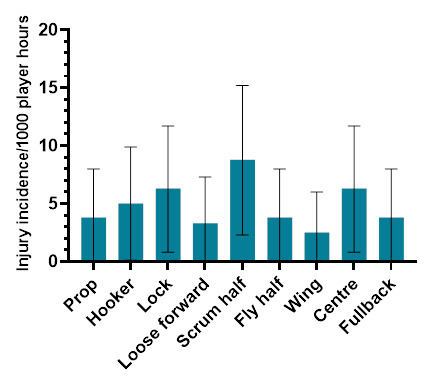
Normalised injury incidence and 95% confidence intervals/1000 player hours per player per position, across the combined 2023 SARU Girls’ Youth Week Tournaments. Missing 2023 data = 1 case.

Figure 13 shows the *normalised* injury incidence per player per position across the two tournaments. In Gu16W, Locks and Centres stood out, and in the Gu18W, Scrumhalf was the standout position. Figure 14 shows the combined *normalised* positional injury rates across both tournaments. In 2023, when combining the data, the Scrumhalf position had the highest *normalised* injury rate across both tournaments. Scrumhalves, when normalised per player, had an incidence of 8.8 (2.3 – 15.2) injuries/1000 player hours (Figure 14).

**Figure:**
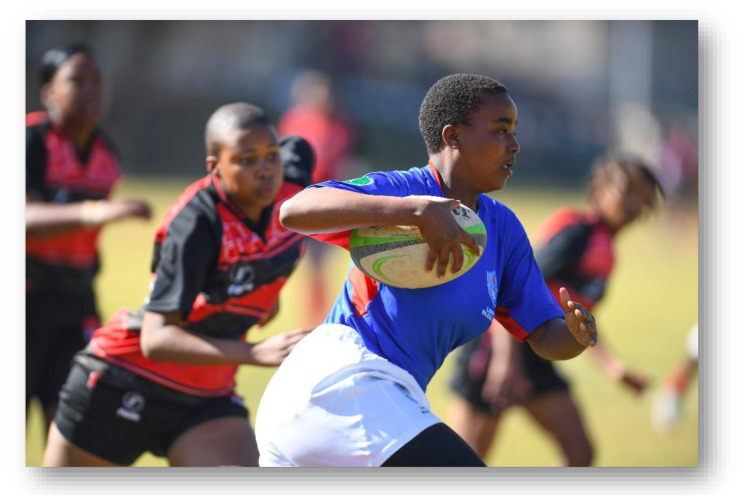

**Figure:**
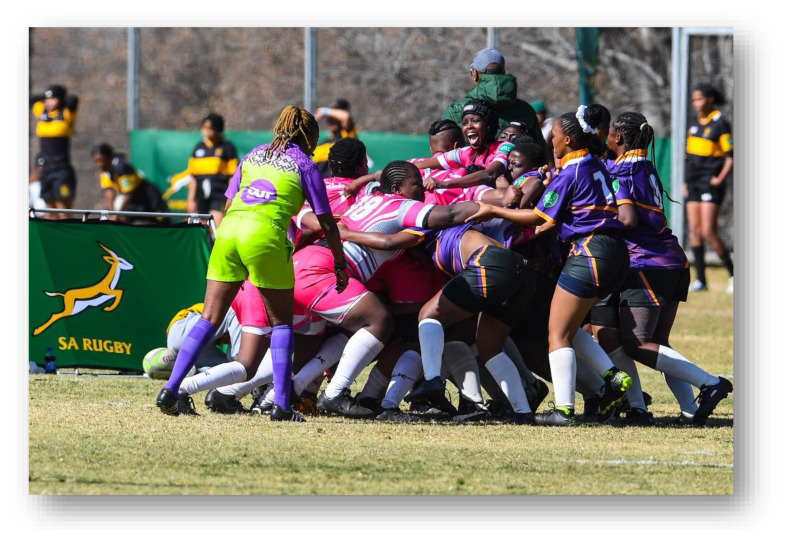

## Concussion

In 2023, there were a total of thirteen concussions (n = 13), keeping in mind that the first day’s Gu16W matches were cancelled due to snow. This translates to an incidence rate of 16 (7 – 25) concussions/1000 player hours and roughly *one concussion for every two matches played*. The concussion number and incidence rate decreased substantially from 2022, where it was last recorded at 29 (19 – 39) concussions/1000 player hours.

The tournament with the highest concussion incidence rate was Gu16W, with 21 (3 – 39) concussions/1000 player hours (Table 8). There were no significant differences between the two tournaments.

**Figure 15:**
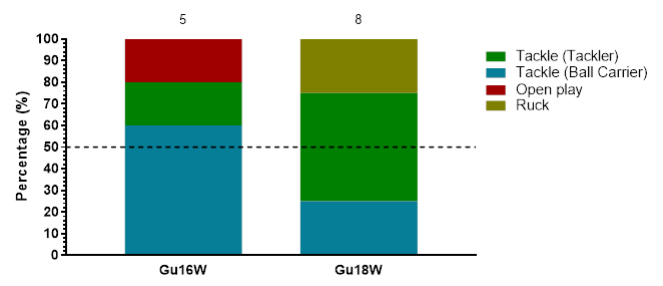
Proportion of concussions caused by the different injury events at the 2023 SARU Girls’ Youth Week Tournaments (n = 13 concussions; Gu16W = 5, Gu18W = 8). (The number above each bar represents the total number of concussions for that tournament). Missing 2023 data = 0 cases.

Tackling (39%, n = 5) and *Being Tackled* (39%, n = 5) contributed to the most concussions in 2023 (Figure 15). Figure 15 displays the proportion of concussions and their different injury-causing events across the two tournaments in 2023.

**Figure 16:**
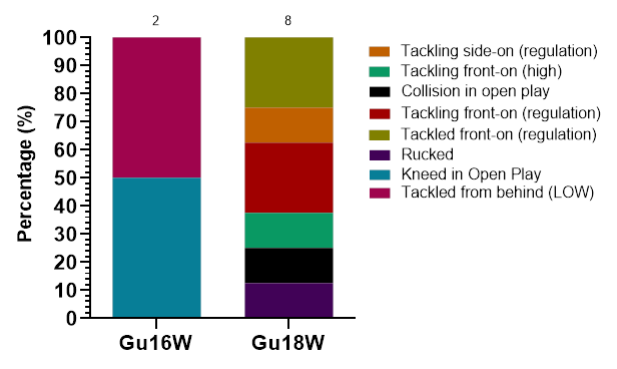
Proportion of concussions caused by the different injury event mechanisms at the 2023 SARU Girls’ Youth Week Tournaments (The number above each bar represents the total number of concussions for that tournament). Missing 2023 data = 3 cases (Gu16W = 3).

*Tackling front-on (regulation)* and *Being Tackled front-on (regulation)* accounted for 31% of all concussions, making the front-on tackle the most prominent singular event causing concussions for the combined tournament data (Figure 16). In Gu18W, *Tackling front-on (regulation)* and *Being Tackled front-on (regulation)* each contributed to 2 of the 8 cases recorded in 2023. Furthermore, *Tackled from behind (LOW)* and *Kneed in Open Play* in Gu16W, contributed to 2 of the 5 cases (Figure 16). Three of the five cases had missing data on the specifics.

**Figure 17:**
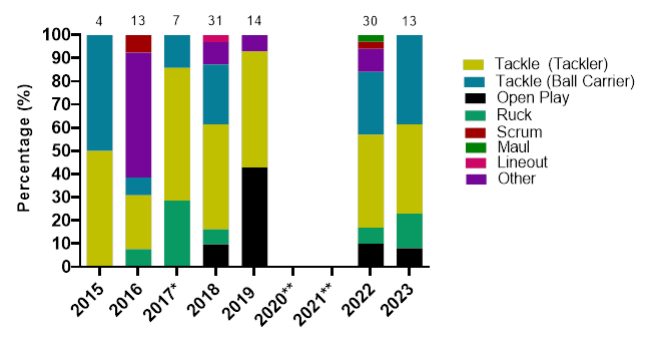
Proportion of concussions caused by the different injury events from 2015 to 2023 at the SARU Girls’ Youth Week Tournaments. (The number above each bar represents the total number of concussions for that year). *No Gu16W tournament was held in 2017. Missing 2023 data = 0 cases. **No Gu16W and Gu18W tournaments were held in 2020 and 2021 due to COVID-19 restrictions.

Figure 17 displays the proportion of concussions caused by the different event mechanisms from 2015 to 2023. The *Tackle* event was the most prominent cause of concussions between 2015 and 2023, contributing to 65% of all concussions: 39% to the Tackler, 26% to the Ball Carrier. This could be due to poor tackle and ball carrying techniques. However, further video-analysis investigations are needed to verify and confirm this. Regardless, due to the high proportion of contributions towards all injuries and concussions, it is apparent that *tackles* and *ball carries* remain the primary injury-causing events and need to be given more attention in the training and preparation of junior South African female rugby players.

The absolute number of concussions increased sizably between 2019 and 2022. The number and rate of concussions in 2023 decreased from the high values recorded in 2022.

**Figure 18:**
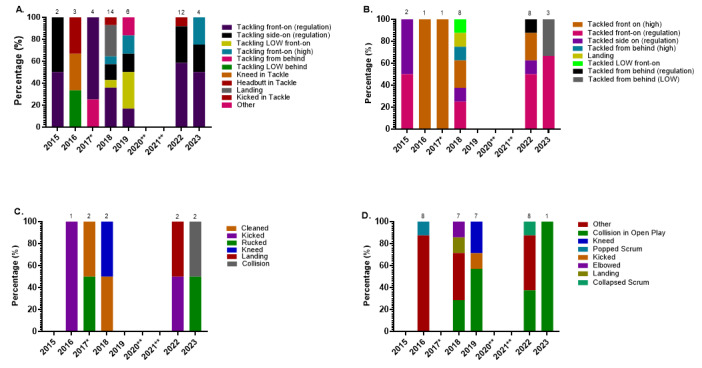
Proportionate breakdown of concussions caused by the various injury-causing mechanisms at the 2015 to 2023 SARU Girls’ Youth Week Tournaments. (The number above each bar represents the total number of concussions for that year in each graph category). **A**. Tackler-related concussion mechanisms **B**. Ball Carrier-related concussion mechanisms **C**. Ruck-related concussion mechanisms. **D**. Remaining concussion mechanisms. *No Gu16W tournament was held in 2017. Missing 2023 data = 3 cases. **No Gu16W and Gu18W tournaments were held in 2020 and 2021 due to COVID-19 restrictions.

Figure 18 displays the proportionate breakdown of concussions resulting from the different injury-causing mechanisms over the nine years.

Between 2015 and 2023, 42% of Tackler-related concussions (Figure 18A) were caused by *Tackling front-on (regulation)*, 39% of Ball Carrier-related concussions (Figure 18B) were caused by *Being Tackled front-on (regulation)*, and 22% of Ruck-related concussions (Figure 18C) were caused by each of the following mechanisms in the breakdown contest, i.e., being *Kicked, Rucked* and *Cleaned out*.

**Figure 19:**
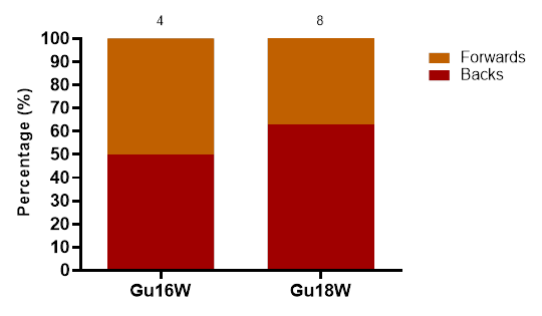
Proportionate breakdown of concussions for forwards and backs at the 2023 SARU Girls’ Youth Week Tournaments (the number above the bar represents the total number of concussions per category for that tournament). Missing 2023 data = 1 case.

Concussions in 2023 were slightly higher in backs than in forwards. In Gu16W, the concussions were evenly distributed. At the Gu18W, 63% of concussions were sustained by backs. (Figure 19).

**Figure 20:**
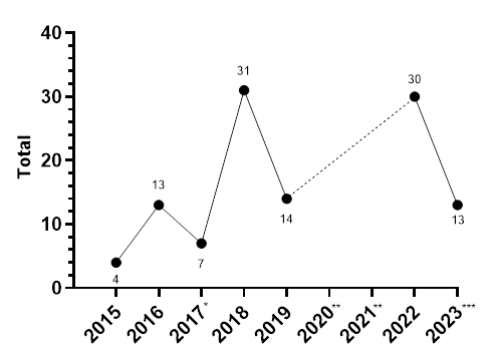
Total number of concussions per year at the SARU Girls’ Youth Week Tournaments from 2015 to 2023. (The number above each data point represents the total number of concussions for that year). *No Gu16W tournament was held in 2017. Missing 2023 data = 0 cases. **No Gu16W and Gu18W tournaments were held in 2020 and 2021 due to COVID-19 restrictions. ***Day 1 of the Gu16W 2023 was cancelled due to snow.

**Figure 21:**
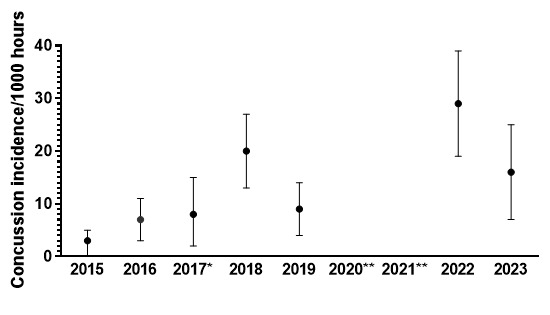
Concussion incidence rates and 95% confidence intervals/1000 player hours per year at the SARU Girls’ Youth Week Tournaments from 2015 to 2023. Missing 2023 data = 0 cases. *No Gu16W tournament was held in 2017. **No Gu16W and Gu18W tournaments were held in 2020 and 2021 due to COVID-19 restrictions.

**Figure 22:**
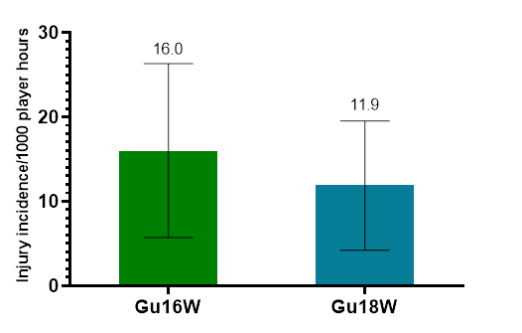
Concussion incidence rates and 95% confidence intervals/1000 player hours per SARU Girls’ Youth Week tournament from 2015 to 2023. No Gu16W tournament was held in 2017. No Gu16W and Gu18W tournaments were held in 2020 and 2021 due to COVID-19 restrictions. The number above each bar represents the average concussion incidence/1000 player hours for the combined time-period of injury surveillance.

Figure 20 illustrates the total number of concussions from 2015 to 2023 for the SARU Girls’ Youth Week Tournaments. The corresponding concussion rate over the same period is shown in Figure 21. The total number of concussions increased sharply from 2019 to 2022, but then decreased in 2023 to a concussion number similar to that of 2019. Keeping in mind that Day 1 of the Gu16W was cancelled due to snow, a similar pattern remained apparent in the incidence of concussion, which normalises the data for player exposure. The spike in 2022 could be attributed to the COVID-19 restrictions in 2020 and 2021; in 2023, these might have decreased back to the original 2018 rates. Players were unable to train, be coached, or compete during COVID-19, which may be a contributing cause to the increased concussion numbers and rates seen in 2022. Alternatively, the interventions provided to the Provincial Unions because of the previous 2022 report, i.e., requesting the provincial Unions to introduce the ‘Preparation for Contact’ and ‘Contact Confident’ Modules to their players at least 8 weeks prior, might have assisted in improving the concussion profile of these tournaments. It will be interesting to see if this is maintained in the next year. When combining all concussions over time per tournament (2015 to 2023), concussions and concussion rates tend to be lower at the Gu18W when compared to the Gu16W (Figure 22). However, these differences are not significant.

**Figure:**
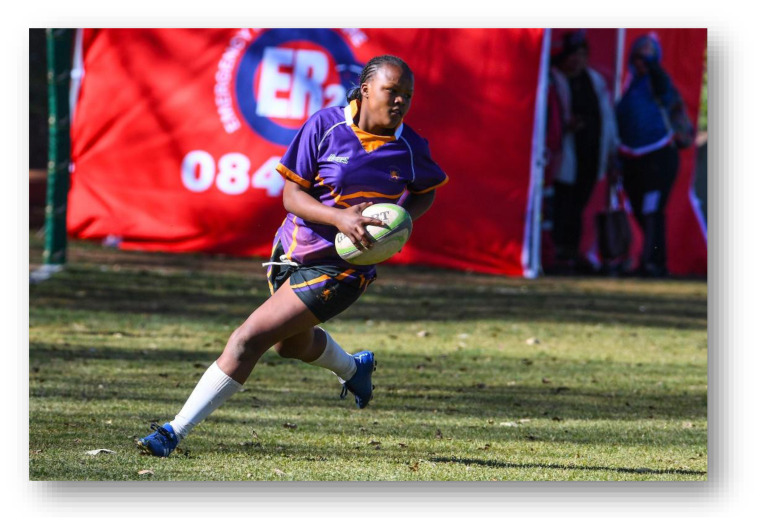

**Figure 23:**
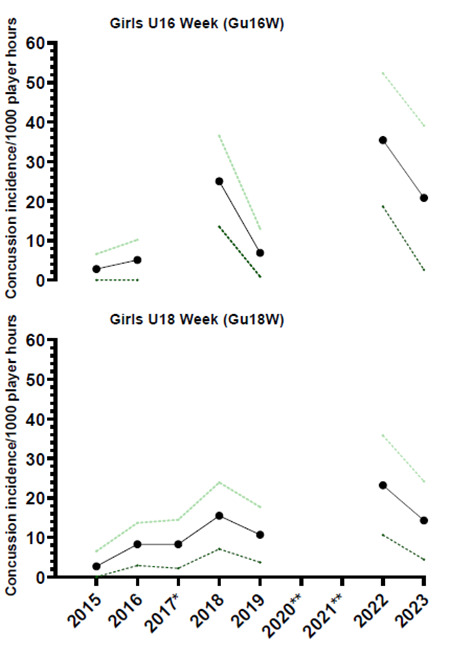
Concussion incidence and 95%CI for each SARU Girls’ Youth Week tournament from 2015 to 2023. *No Gu16W tournament was held in 2017. **No Gu16W and Gu18W tournaments were held in 2020 and 2021 due to COVID-19 restrictions.

The incidence of concussions during the Gu16W and Gu18W tournaments follows a similar trend to the injury rates. Concussion incidence rates gradually increased from around 2015 to 2018. However, from 2018 to 2019, there was a notable decrease in concussion incidence, followed by a reversal and large increase in concussion incidence in 2022. Positively, though, there is a decrease from 2022 to 2023 (Figure 23).

In Figure 23, there is no clear pattern of concussions for the Gu16W between 2015 and 2019. Concussions increased sharply from 2019 to 2022, followed by a decrease in 2023. There was no Gu16W tournament in 2017, 2020 and 2021, so the trendline could not be accurately calculated and was therefore excluded.

Similarly, in the Gu18W, the trendline could not be calculated accurately; therefore, it was also excluded. In Gu18W, there was a sizable increase in concussions in 2018, followed by a decline in concussions in 2019. In 2022, there was a notable increase in concussion incidence. However, there was a decrease in concussion incidence from 2022 to 2023.

**Figure:**
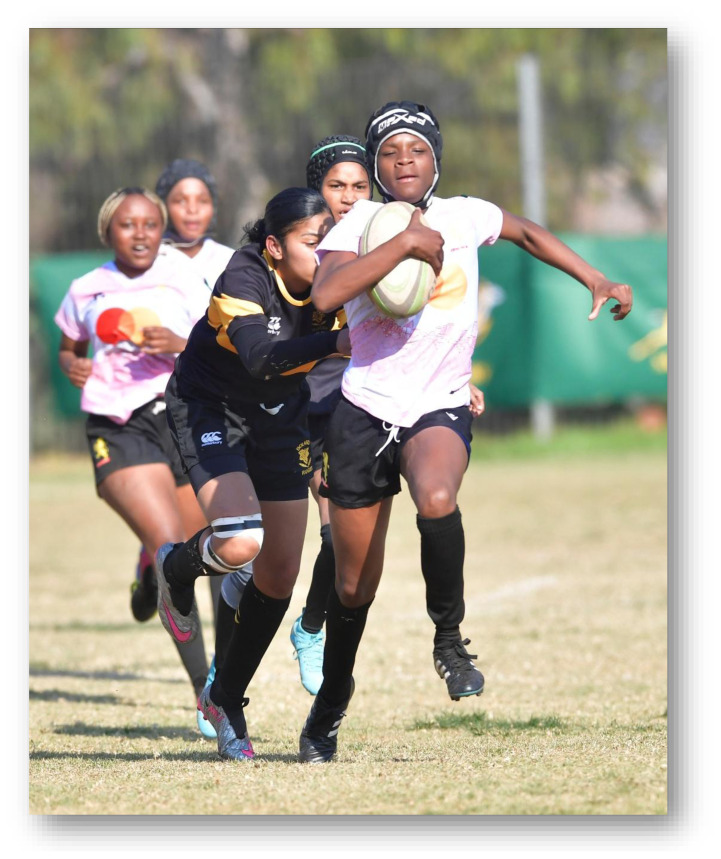

## Take-home messages

1. The current format involves each team playing two matches per week, with a full rest day in between. This format should remain in place until the Girls Youth Game at schools and youth clubs in South Africa becomes more established.
2. The tackle event causes most of the time-loss injuries and concussions.
3. Although more research is required to substantiate this, there is a large possibility that these injuries are due to poor tackle and ball carrying techniques.
4. It is important to continue increasing the confidence and proficiency of young female rugby players in contact situations, as this contributes significantly to injuries and concussions. Developing the basic contact-safety skills, techniques, and required levels of proficiency through progressive and tailored training is crucial for junior South African female rugby players. This will help minimise concussions and other rugby-related injuries. It is important to prioritise such training to ensure that the players can safely tackle and carry the ball into contact.
5. Continue using the “Preparation for Contact” and “Contact Confident” programmes, freely accessible on the World Rugby education platform and MyBokSmart, to support the training and contact preparation of junior South African female rugby players. Although not directly confirmed, it may have had a positive impact on concussion rates in 2023.

## Figures and Tables

**Table 1:** Number and incidence (95% CI)/1000 player hours of Medical Attention and Time-Loss injuries in the 2023 SARU Girls’ Youth Week tournaments.

	Medical Attention Injuries		Time-Loss Injuries	

	Number	Incidence	Number	Incidence
**Gu16W**	30	125 (80 – 170)	21	88 (50 – 125)
**Gu18W**	47	84 (60 – 108)	35	63 (42 – 83)

***Combined Total***	***77***	***96 (75 – 118)***	***56***	***70 (52 – 88)***

**Table 2:** Number of Medical Attention and Time-Loss Injuries. Data are expressed per match and per hour played in the 2023 SARU Girls’ Youth Week tournaments.

Tournament	Number of matches	Match duration (mins)	Medical Attention (injuries/match)	Time-Loss (injuries/match)	Medical Attention (injuries/hour)	Time-Loss (injuries/hour)
**Gu16W**	8	60	3.8	2.6	3.8	2.6
**Gu18W**	16	70	2.9	2.2	2.5	1.9

***Combined Tournament Average***	***12***	***65***	***3.3***	***2.4***	***3.1***	***2.3***

**Table 3:** Injury incidence (95% CI)/1000 player hours of Time-Loss injuries to the Tackler and Ball Carrier roles (within the Tackle) and Open Play, for the 2023 SARU Girls’ Youth Week tournaments.

Tournament	Tackler	Ball Carrier	Ruck
**Gu16W**	29 (8 – 51)	38 (13 – 62)	13 (0 – 27)
**Gu18W**	29 (15 – 43)	18 (7 – 29)	13 (3 – 22)

***Combined total***	***29 (17 – 41)***	***24 (13 – 34)***	***13 (5 – 20)***

**Table 4:** Injury incidence (95% CI)/1000 player hours of Time-Loss injuries at the 2023 SARU Girls’ Youth Week tournaments grouped as Central Nervous System (CNS), Joint/Ligament, and Muscle/Tendon injuries.

Tournament	CNS	Joint/Ligament	Muscle/Tendon
**Gu16W**	21 (3 – 40)	8 (0 – 20)	13 (0 – 27)
**Gu18W**	14 (4 – 24)	18 (7 – 29)	7 (0 – 14)

***Combined Total***	***16 (7*** ** – 25** ***)***	***15 (7 – 24)***	***9 (2 – 15)***

**Table 5:** Proportion (%) and incidence (95% CI)/1000 player hours of Time-Loss injuries, grouped by body location, in the 2023 SARU Girls’ Youth Week tournaments.

	Proportion of injuries (%)	Incidence (95% CI)/1000 player hours
**Lower Body**	39	**28 (16 – 39)** *
**Head and Neck**	30	21 (11 – 31)
**Upper Body**	18	13 (5 – 20)
**Trunk**	13	9 (2 – 15)

* Significantly higher than Trunk injuries

**Table 6:** Injuries grouped according to the IOC recommended categories of Tissue and Pathology types for the 2023 SARU Girls’ Youth Week tournaments. Missing 2023 data for mean time loss = 5 cases.

Tissue	Injuries	Incidence	Mean time loss

*Pathology*	*n*	*Injuries per 1000 hours (95% CI)*	*Days (95% CI)*
**Muscle/Tendon**	**7**	**9 (3 – 15)**	-
*Muscle injury*	6	8 (2 – 14)	-
*Tendinopathy*	1	1 (0 – 4)	-
**Nervous**	**13**	**16 (7 – 25)**	**19 (18 – 20)**
*Brain/Spinal cord injury*	13	16 (7 – 25)	19 (18 – 20)
**Joint Sprain/Ligament tear**	**12**	**15 (7 – 24)**	**25**
**Bone**	**10**	**13 (5 – 20)**	**14 (10 – 19)**
*Fracture*	4	5 (0 – 10)	7
*Bone stress fracture*	2	3 (0 – 6)	-
*Bone contusion*	4	5 (0 – 10)	18 (14 – 22)
***Cartilage/Synovium/Bursa***	3	**4 (0 – 8)**	**45**
*Cartilage injury*	3	4 (0 – 8)	45
**Superficial tissue/skin**	**6**	**8 (2 – 14)**	1
*Contusion (superficial)*	5	6 (1 – 12)	1
*Laceration*	1	1 (0 – 4)	-

***TOTAL***	**56**	***70 (52 – 88)***	**21 (18 – 23)**

Where n = 1, mean Time-Loss reflects the total Time-Loss days. Estimated severity for Time-Loss was used from data provided by the Tournament Medical Doctors at the venue when real-time severity was not able to be determined.

**Table 7:** Number of injuries and injury rates (95% CI)/1000 exposure hours of players who started the match and those who came on as substitutions in the 2023 SARU Girls’ Youth Week tournaments. Missing 2023 data = 0 cases.

	Started match		Substitution	

	Number	Incidence	Number	Incidence
**Gu16W**	21	88 (50 – 125) *	0	0
**Gu18W**	33	59 (39 – 79) *	2	4 (0 – 9)
***Combined Total***	***54***	***68 (50* –*86)*** *	***2***	***3 (0*** ** – 6** ***)***

* Starters were significantly different to Substitutions

**Table 8:** Number and incidence of concussions (95% CI)/1000 player hours at the 2023 SARU Girls’ Youth Week tournaments.

Tournament	Number	Incidence	Number of matches per concussion event
**Gu16W**	5	21 (3 – 39)	2
**Gu18W**	8	14 (4 – 24)	2

***Combined Total***	***13***	***16 (7 – 25)***	***2***

## References

[1] Fuller CW Molloy MG Bagate C Bahr R Brooks JHM Donson H Consensus statement on injury definitions and data collection procedures for studies of injuries in rugby union Br J Sports Med 2007 41 5 328 31 10.1136/bjsm.2006.033282 17452684 PMC2659070

[2] Bahr R Clarsen B Derman W Dvorak J Emery CA Finch CF International Olympic Committee consensus statement: methods for recording and reporting of epidemiological data on injury and illness in sport 2020 (including STROBE Extension for Sport Injury and Illness Surveillance (STROBE-SIIS)) Br J Sports Med 2020 bjsports-2019-101969 10.1136/bjsports-2019-101969 PMC7146946 32071062

[3] Fuller CW A Kinetic Model Describing Injury-Burden in Team Sports Sport Med 2017 47 12 2641 51 10.1007/s40279-017-0746-7 28573403

[4] Schenker N Gentleman JF On judging the significance of differences by examining the overlap between confidence intervals Am Stat 2001 55 3 182 6 [10.1198/000313001317097960]

